# Transformer‐based representation learning and multiple‐instance learning for cancer diagnosis exclusively from raw sequencing fragments of bisulfite‐treated plasma cell‐free DNA

**DOI:** 10.1002/1878-0261.13745

**Published:** 2024-10-08

**Authors:** Jilei Liu, Hongru Shen, Yichen Yang, Meng Yang, Qiang Zhang, Kexin Chen, Xiangchun Li

**Affiliations:** ^1^ Tianjin Cancer Institute, Tianjin's Clinical Research Center for Cancer, National Clinical Research Center for Cancer, Key Laboratory of Cancer Prevention and Therapy, Tianjin Medical University Cancer Institute and Hospital Tianjin Medical University China; ^2^ Department of Maxillofacial and Otorhinolaryngology Oncology, Tianjin's Clinical Research Center for Cancer, Tianjin Medical University Cancer Institute and Hospital Tianjin Medical University China; ^3^ Department of Epidemiology and Biostatistics, Key Laboratory of Molecular Cancer Epidemiology of Tianjin, Tianjin's Clinical Research Center for Cancer, Key Laboratory of Prevention and Control of Major Diseases in the Population Ministry of Education, National Clinical Research Center for Cancer, Key Laboratory of Cancer Prevention and Therapy, Tianjin Medical University Cancer Institute and Hospital Tianjin Medical University China

**Keywords:** cell‐free DNA, early cancer diagnosis, weakly supervised learning

## Abstract

Early cancer diagnosis from bisulfite‐treated cell‐free DNA (cfDNA) fragments requires tedious data analytical procedures. Here, we present a deep‐learning‐based approach for early cancer interception and diagnosis (DECIDIA) that can achieve accurate cancer diagnosis exclusively from bisulfite‐treated cfDNA sequencing fragments. DECIDIA relies on transformer‐based representation learning of DNA fragments and weakly supervised multiple‐instance learning for classification. We systematically evaluate the performance of DECIDIA for cancer diagnosis and cancer type prediction on a curated dataset of 5389 samples that consist of colorectal cancer (CRC; *n* = 1574), hepatocellular cell carcinoma (HCC; *n* = 1181), lung cancer (*n* = 654), and non‐cancer control (*n* = 1980). DECIDIA achieved an area under the receiver operating curve (AUROC) of 0.980 (95% CI, 0.976–0.984) in 10‐fold cross‐validation settings on the CRC dataset by differentiating cancer patients from cancer‐free controls, outperforming benchmarked methods that are based on methylation intensities. Noticeably, DECIDIA achieved an AUROC of 0.910 (95% CI, 0.896–0.924) on the externally independent HCC testing set in distinguishing HCC patients from cancer‐free controls, although there was no HCC data used in model development. In the settings of cancer‐type classification, we observed that DECIDIA achieved a micro‐average AUROC of 0.963 (95% CI, 0.960–0.966) and an overall accuracy of 82.8% (95% CI, 81.8–83.9). In addition, we distilled four sequence signatures from the raw sequencing reads that exhibited differential patterns in cancer versus control and among different cancer types. Our approach represents a new paradigm towards eliminating the tedious data analytical procedures for liquid biopsy that uses bisulfite‐treated cfDNA methylome.

## Graphical abstract

This study introduces DECIDIA, a deep‐learning approach for early cancer diagnosis using bisulfite‐treated cfDNA sequencing fragments. Utilizing transformer‐based representation learning and weakly supervised multiple‐instance learning, DECIDIA accurately detects cancer and predicts cancer types, significantly simplifying data analysis. By offering an end‐to‐end solution for liquid biopsy‐based diagnostics, DECIDIA advances the potential for early, non‐invasive cancer interception.

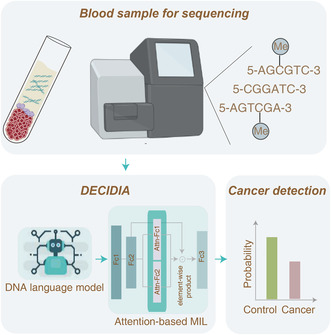

**Abbreviations**
AUROC: area under the receiver operating curve
CCGA: circulating cell‐free genome atlas
cfDNA: cell‐free DNA
CRC: colorectal cancer
HCC: hepatocellular cell carcinoma
NPV: negative predictive value
PCA: principal component analysis
PPV: positive predictive value
ROC: receiver operating curve
SRA: Sequence Read Archive
TCGA: The Cancer Genome Atlas

## 1 Introduction

Plasma cell‐free DNA (cfDNA) testing has been widely studied for early cancer diagnosis [1]. cfDNA is mainly released from cell apoptosis, necrosis, and active secretion [2]. The half‐life of cfDNA in blood is estimated to range from 16 min to 2.5 h [3]. Therefore, cfDNA is seen as a real‐time snapshot manifesting physiological condition of the human body, offering diagnostic information. A number of methods have been proposed for exploiting cfDNA fragmentomics to detect cancer. For instance, GEMINI utilizes the mutation spectrum derived from cfDNA whole‐genome data to detect lung cancer and liver cancer [4]. DELFI is built upon machine learning to integrate multiple fragmentomic features for multiple cancer‐type detection [5]. Statistical methods such as motif diversity score (MDS) [6] and non‐negative matrix factorization [7] were proved to be useful for characterizing cfDNA fragmentomic signatures and liver cancer detection. Additionally, multimodal approaches such as THEMIS [8], CRAG [9], and HIFI [10] have been proposed for early cancer diagnosis by combining cfDNA methylation, chromatin accessibility, and fragmentomic features. Despite achieving high accuracy, these methods often involve complex data analysis steps that are computationally intensive and prone to introducing bias and batch effect [11].

DNA methylation alterations are known to occur early in tumorigenesis and more sensitive in detecting cancer compared to genomic DNA alterations, offering valuable insights into cancer development and cancer origin localization [12, 13]. Bisulfite sequencing enables single‐base resolution for characterizing DNA methylation. The core idea of bisulfite sequencing is that bisulfite treatment can deaminate unmethylated cytosine to uracil while methylated cytosine remains unchanged. Profiling plasma cfDNA by bisulfite sequencing is a promising solution for developing blood‐based early cancer diagnosis [14, 15]. The Circulating Cell‐free Genome Atlas (CCGA) project is one of the representative endeavors devoted to developing blood‐based early cancer diagnosis by profiling plasm cfDNA with bisulfite sequencing. Three studies from the CCGA group reported that the methylation patterns of plasma cfDNA are able to precisely detect multiple cancer types at earlier stage [16, 17]. Meanwhile, Xu and colleagues also reported high sensitivity and specificity in the diagnosis of colorectal cancer and hepatocellular carcinoma by profiling cfDNA methylation via targeted‐bisulfite sequencing [18, 19].

However, some of these methods such as HIFI and THEMIS rely on sequencing alignment for quantification of methylation abundance, followed by multiple data analysis procedures that are time‐consuming and error‐prone—the more the steps the higher the error rate due to error accumulation [20]. For instance, alignment is time‐consuming and one of the major steps for most of these methods. However, reads that are useful for cancer detection but cannot be mapped onto the reference genome are discarded. Additionally, biases in methylation profiling of cfDNA are aggravated due to the fragmented nature, low concentration of cfDNA, and high background noise [21]. This underscores the need for innovative approaches to overcome these challenges and improve the accuracy and reliability of diagnostics.

Transformer‐based pre‐trained language models achieve excellent performance in many natural language understanding such as translation and semantic analysis [22]. This solution has also been adopted in learning the feature representation of DNA and protein sequences. For example, deep language models pre‐trained on human reference genome can capture the global and transferable representation of the genomic DNA sequence and achieve state‐of‐the‐art performance at characterization of promoters, splice sites, and transcription factor binding sites [23]. Multiple‐instance learning is a type of weakly‐supervised deep‐learning algorithm [24] that is useful for classification in the scenario where only label of the individual is available but the labels of the instances of that individual are not available. By coupling with the attention‐based mechanism, this weakly supervised learning approach is able to identify the instances that are most relevant for classification [25, 26].

In this study, we present a deep‐learning‐based approach for early cancer interception and diagnosis called DECIDIA exclusively from the raw sequencing reads of bisulfite‐treated plasma cfDNA fragments. DECIDIA consists of development of a transformer‐based module for learning the features of DNA sequences and application of multiple‐instance learning for aggregating multiple sequencing reads for classification. We systematically evaluated the performance of DECIDIA for cancer diagnosis and cancer type prediction on a curated dataset of 5389 samples that consist of colorectal cancer (CRC, *n* = 1574), hepatocellular cell carcinoma (HCC, *n* = 2140), lung cancer (*n* = 654) and non‐cancer control (*n* = 1980) subjected to targeted‐bisulfite sequencing of plasma cfDNA. We demonstrated that DECIDIA is robust and efficient for varying number of sample size and sequencing reads. DECIDIA is quite simple in essence in that it directly uses raw sequencing reads instead of methylation abundance. DECIDIA eliminates the tedious data analytical procedures required in developing early cancer diagnosis tools using plasma cfDNA methylation. DECIDIA directly models the relation between raw sequencing cfDNA reads and diagnostic labels in a single step in an end‐to‐end manner, thus simplifying data analysis procedures and reducing accumulated errors. It will facilitate the development of cfDNA‐based diagnostic tools.

## 2 Materials and methods

### 2.1 Data collection and preprocessing

We collected the sequencing data of 5389 individuals that were subjected to bisulfite sequencing of plasma cfDNA from The Sequence Read Archive (SRA) database. The Accession Numbers are PRJNA574555 [18], PRJNA360288 [19], PRJNA383358 [27] and PRJNA383370 [27]. Detailed information for these datasets is provided in Table S1. PRJNA574555 contains 801 patients with colorectal cancer (CRC) and 1021 healthy individuals (denoted as CRC dataset). PRJNA360288 consists of 1181 patients with hepatocellular carcinoma (HCC) and 959 healthy controls (denoted as HCC dataset). PRJNA383358 includes 773 patients with CRC and no cancer‐free controls. PRJNA383370 has 654 patients with lung cancer and no cancer‐free controls. The CRC dataset was used to develop the deep‐learning model while the HCC dataset was used to independently validate the model. Cancer samples from the CRC and HCC datasets, along with samples from PRJNA383358 and PRJNA383370 were used for the development of cancer type classification model. We randomly obtained 1000 reads from each individual, which account for 0.03–0.1% of total sequencing reads. We used the Python package *pysam* to read the Fastq files and utilized the *get_quality_array* function to obtain base quality scores. We dropped reads with ambiguous base or average base quality score < 30. Both R1 and R2 reads were included in this analysis. All data have been subjected to adapter trimming.

### 2.2 DECIDIA for cancer diagnosis

We used the CRC dataset (PRJNA574555) for developing cancer diagnostic model. We adopted the 10‐fold Monte Carlo cross‐validation. Within each cross‐validated fold, we randomly partitioned the total CRC dataset into a training set (*n* = 1000), validation set (*n* = 200) and testing set (*n* = 622). For downsample analysis, the iterated numbers of sequencing reads (< 1000) and samples (< 1000) were randomly taken from the initially selected 1000 reads or samples in the corresponding fold. We used the training set to train the model and validation set to choose the best checkpoint and subsequently evaluated its performance on the testing set. The model with the highest accuracy in the 10‐fold cross‐validation was used for downstream analysis. In addition, we used the HCC dataset (PRJNA360288) as an external testing set.

### 2.3 DECIDIA for cancer type detection

We curated a dataset of 3409 cancer samples from PRJNA574555, PRJNA360288, PRJNA383358, and PRJNA383370 including colorectal cancer (CRC, *n* = 1154), HCC (*n* = 1181) and lung cancer (*n* = 654). We adopted 10‐fold Monte Carlo cross‐validation. Within each cross‐validated fold, we split this dataset into training (*n* = 2308), validation (*n* = 300) and testing (*n* = 800) sets. We used the training set to train the model and validation set to choose the best checkpoint and subsequently evaluated its performance on the testing set. The model with the highest accuracy in the 10‐fold cross‐validation was used for downstream analysis.

### 2.4 Preprocessing of DNA sequence

We constructed a dictionary ***D*** with seven tokens, i.e., ***D*** = {<*s*>, <*e*>, <*pad*>, A, C, G, T}. According to previous study [23], we added <*s*> and <*e*> to the beginning and end of the input DNA sequence, respectively. The <*pad*> token is used to pad the input DNA sequence to a fixed length if its length is less than a predefined value. The input DNA sequence is truncated if its length exceeds the predefined value. In this study, we set the predefined DNA sequence length to 75. For an input DNA sequence ***S***, e.g., ***S*** = GAGTACG, it was first converted into a character vector ***B*** = {<*s*>, G, A, G, T, A, C, G, <*e*>}, subsequently it was converted into the integer vector of indices according to the dictionary ***D***, i.e., ***X*** = {0, 5, 3, 5, 5, 3, 4, 5, 1}. ***X*** is the input fed to DECIDIA.

### 2.5 Feature representation learning of DNA sequence

We used the autoregressive language model to learn the feature representation of DNA sequences. Specifically, for an input DNA sequence of length *n* bases $S=\left\{b_{1},b_{2},b_{3}\ldots ,b_{n}\right\}$, the purpose of autoregressive modeling is to predict the base *b*_*i*_ in the context of all its preceding bases, i.e., $b_{1},b_{2},b_{3}\ldots ,b_{i-1}$. The standard language modeling objective $ℒ\left(S\right)=\sum_{i}\log P\left(b_{i}|b_{1},b_{2},b_{3}\ldots ,b_{i-1};\theta \right)$ is used to maximize the likelihood. θ are the parameters of the autoregressive model that is used to model the conditional probability. We used the human reference genome to train the autoregressive model by splitting it into sequences of 100 base pairs. All genomic regions of the human genome reference were included in this training. The autoregressive model was trained independently of the raw bisulfite sequencing data, which was not considered in its training. In our study, we used a single‐layer transformer decoder for modeling the ‘language grammar’ of DNA sequence. This language model employs a transformer block followed by softmax to output the distribution over each nucleotide. The transformer block consists of multi‐headed self‐attention operation and position‐wise feed‐forward layer. Let *W*_*e*_ denote the nucleotides embedding matrix and *W*_*p*_ the position embedding matrix. This language model can be formulated as [28]:

$$\begin{array}{c} h_{0}={\mathrm{SW}}_{e}+W_{p}\\ h_{1}=\text{transformer}\_\text{block}\left(h_{0}\right)\\ P\left(b\right)=\text{softmax}\left(h_{1}W_{e}^{T}\right) \end{array}$$

The self‐attention head *i* applies the scale dot‐product attention to map a query *Q*_*i*_ and a set of key‐value pairs (*K*_*i*_ and *V*_*i*_) to output. *Q*_*i*_, *K*_*i*_ and *V*_*i*_ are projections from the input embedding layer that encodes the input DNA sequence. Let *d*_*k*_ be the dimension of query and key values. Self‐attention is defined as:

$$\text{selfatten}\left(Q_{i},K_{i},V_{i}\right)=\text{softmax}\left(\frac{Q_{i}K_{i}^{T}}{\sqrt{d_{k}}}\right)V_{i}$$

Multi‐headed self‐attention is simply a concatenation of multiple self‐attention heads:

$$\text{MultiHead}\left(Q,K,V\right)=\text{Concat}({\text{selfatten}}_{1},\ldots ,{\text{selfatten}}_{4})W^{o}$$

where *W* ^*o*^ is the projection parameter. Position‐wise feed‐forward layer is defined as:

$$\mathrm{FFN}=\max\left(0,xW_{1}+b_{1}\right)W_{2}+b_{2}$$

where *W*_1_ and *W*_2_ are weight matrices and *b*_1_ and *b*_2_ are the bias.

### 2.6 Attention‐based multiple‐instance classifier

In the setting of multiple‐instance learning, a sample is considered as a bag and reads of that sample are instances. For a given sample with *k* reads, we can obtain a feature matrix, denoted as:

$${\left[X_{1},X_{2},\ldots ,X_{k}\right]}^{T}$$

*X*_*i*_ is the feature for the *i*th read outputted from the DNA sequence language model.

A two‐layer fully connected neural network is used to transform the learned DNA sequence feature into latent vector *h*_*i*_.

$$h_{i}=\text{ReLU}\left(W_{2}\left(\text{ReLU}\left(W_{1}X_{i}+b_{1}\right)\right)+b_{2}\right)$$

*W*_1_, *W*_2_, *b*_1_ and *b*_2_ are parameters and ReLU is the activation function. The attention weight *a*_*i*_ for *h*_*i*_ is defined as [25]:

$$a_{i}=\frac{\exp\left(\tan h\left(Vh_{i}\right)⊙\text{sigmoid}\left(Uh_{i}\right)\right)}{\sum_{j=1}^{k}\exp\left(\tan h\left(Vh_{j}\right)⊙\text{sigmoid}\left(Uh_{j}\right)\right)}$$

where *V* and *U* are weight parameters; tan*h* and sigmoid are activation functions. Attention pooling was applied to obtain the sample‐level features:

$$Z=H^{T}A$$

where *A* = {*a*_1_, *a*_2_, *a*_3_, …, *a*_*k*_}, *H* = {*h*_1_, *h*_2_, *h*_3_, …, *h*_*k*_}. A fully connected layer parameterized as *W*_3_ and *b*_3_ followed by softmax was used to transform the sample‐level features into probabilities:

$$p=\text{softmax}\left(W_{3}Z+b_{3}\right)$$

### 2.7 Construction of sequence patterns

The last row of each attention matrix (denote as *a*_*i*_) represents the association of each nucleotide in the input DNA sequence on the representation of the input sequence. For each sample, we retrieved the top 100 reads according to the important scores derived from the aforementioned multiple‐instance classifier. We respectively summed up *a*_*i*_ for reads belonging to different phenotypes stratified by A, C, G and T nucleotides at different positions.

### 2.8 Training scheme

The feature representation model is a decoder‐only transformer [29] with a single decoder block whose hidden dimension is set to 384 and number of attention heads is set to 4. Feature representation model was pre‐trained with a batch‐size of 4096 for 40 epochs. We used AdamW [30] with β1 = 0.9, β2 = 0.999, weight decay of 0.01 and an initial learning rate of 1e‐4. The learning rate is warmed up for 3 epochs and then decays to 0 following a cosine schedule [31]. The weakly supervised classifier was trained with a batch‐size of 1 sample for 100 epochs. We use the cross‐entropy loss as the objective function and AdamW optimizer with β1 = 0.9, β2 = 0.999 and weight decay of 1e‐5. Learning rate was set to a constant value of 2e‐5. All models were trained with pytorch (version 1.7.1, Meta Platforms, Inc., Menlo Park, CA, USA) and transformers (version 4.10.0, https://github.com/huggingface/transformers) on nvidia dgx A100 (NVIDIA Corp., Santa Clara, CA, USA).

### 2.9 Details of the data process and experimental properties of datasets

The four datasets used in this study come from different studies but share the same library preparation and sequencing strategy. Detailed data processing and experimental properties of the datasets are provided below.

#### 2.9.1 Patient information

The study involved cancer patients and healthy controls from China. Patients aged 20–85 years, without other malignant tumors, willing to provide blood samples, and who consented to participate were included. Controls were healthy individuals undergoing routine body examinations. Blood samples were collected through venipuncture, and plasma samples were obtained by centrifugation and stored at −80 °C for later cfDNA extraction.

#### 2.9.2 Cell‐free DNA extraction

Plasma samples were stored at −80 °C until use. It was determined that to reliably achieve over 20 000 unique reads per sample, at least 1.5 mL of plasma was required. This volume typically yielded more than 10 ng of cfDNA, which provided at least 140 copies of detected amplicons in digital droplet PCR assays (median 398 copies). For consistent DNA methylation measurement, a minimum of 15 ng of cfDNA per 1.5 mL of plasma was used. The EliteHealth cfDNA extraction kit was employed, following the manufacturer's instructions.

#### 2.9.3 Bisulfite conversion

10 ng of genomic or cfDNA was converted to bisulfite‐treated DNA (bis‐DNA) using the EZ DNA Methylation‐Lightning™ Kit (Zymo Research Corp., Irvine, CA, USA), as per the manufacturer's protocol. The resulting bis‐DNA had a size distribution of approximately 200–3000 bp, peaking around 500–1000 bp. The conversion efficiency was confirmed to be over 99.8% by deep‐sequencing and analyzing the conversion ratio of C to T in non‐CG dinucleotides.

#### 2.9.4 DNA methylation analysis

Unsupervised hierarchical clustering identified the top 1000 markers distinguishing specific cancer from normal samples on the TCGA datasets. For capture‐sequencing, 1000 molecular inversion (padlock) probes were designed. The technique, based on published methods with modifications, used shorter probes (around 80 bp) for the more fragmented bisulfite‐converted cfDNA (~ 120 bp in size). A library‐free protocol was used to reduce costs and preparation time, and an efficient bioinformatics pipeline facilitated accurate methylation quantification and genotyping.

#### 2.9.5 Probe design and validation

Probes were designed using ppdesigner software (University of California, San Diego, La Jolla, CA, USA), targeting a 100 bp region centered around the CpG marker. A 6‐bp unique molecular identifier (UMI) was included for distinguishing individual capture events. Probes were synthesized as separate oligonucleotides by IDT and purified using Qiagen columns. Sequencing data from pilot experiments revealed efficiency differences among probes; thus, relative efficiencies were calculated, and probe concentrations were adjusted to achieve 85% coverage uniformity at greater than 0.5× average coverage.

#### 2.9.6 Bis‐DNA capture and sequencing

10 ng of bis‐DNA was mixed with padlock probes in reactions containing Ampligase buffer, denatured, and then slowly cooled for hybridization. The gap between annealed arms was filled with PfuTurboCx polymerase and Ampligase, followed by exonuclease treatment to degrade single‐stranded DNA. Circular capture products were amplified via PCR, incorporating barcodes and Illumina sequencing adaptors. Amplification cycles were optimized, and libraries were size‐selected to exclude incomplete captures. The libraries were verified for purity and concentration before sequencing on MiSeq and HiSeq2500 platforms.

### 2.10 Statistical analysis and software

We used ROC curve, accuracy, sensitivity, specificity, positive predictive value (PPV), and negative predictive value (NPV) to measure the performance of DECIDIA. The ROC curve was created by plotting sensitivity against specificity. The 95% confidence intervals for accuracy, sensitivity, specificity, PPV, and NPV were calculated by Clopper–Pearson method [32]. We plotted the ROC curve and calculated AUC with r package pROC (version 1.18.0) and multiroc (version 1.1.1). Statistical analysis was conducted with r software (version 4.1.0) and caret package (version 6.0‐90).

### 2.11 Ethics statement

This study was approved by the Institutional Review Board (approval number: EK20240105) of Tianjin Medical University Cancer Institute and Hospital. Informed consent from patients is not applicable and exempted as this study use publicly available data.

## 3 Results

### 3.1 Overview of DECIDIA

**Fig. 1:**
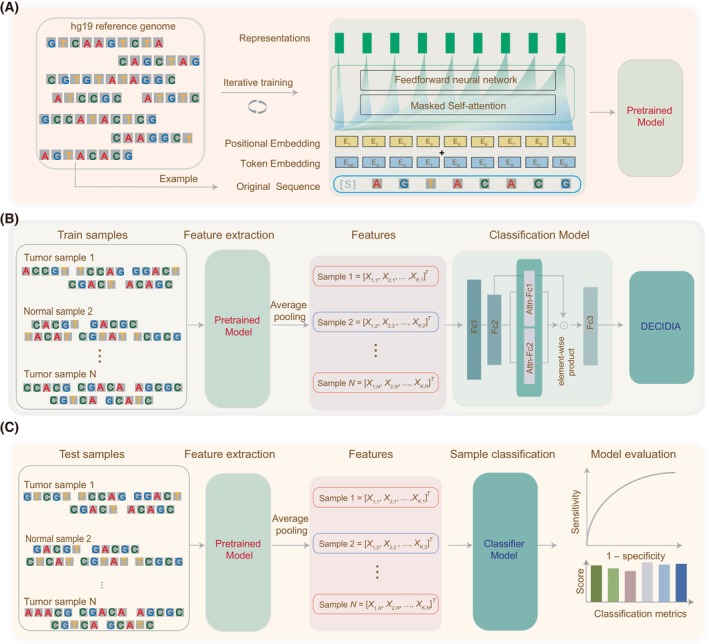
A flowchart depicting the development and evaluation of DECIDIA. (A) Development of an autoregressive language model on human reference genome for learning feature representations of DNA sequences. (B) Development of a multiple‐instance learning classifier with attention mechanism for cancer diagnosis and cancer type classification. (C) Performance evaluation on testing sets.

Fundamentally, DECIDIA consists of two components including the development of a feature representation learning model for sequencing reads and construction of an attention‐based multiple‐instance learning model for sample‐level classification by aggregating multiple reads from that sample. We employed the autoregressive language model [28] for learning DNA sequence feature. For an input DNA sequence $S=\left\{b_{1},b_{2},b_{3}\ldots ,b_{n}\right\}$, the purpose of autoregressive modeling is to predict the base $b_{i}$ in the context of all its preceding bases, i.e., $b_{1},b_{2},b_{3}\ldots ,b_{i-1}$. Data used to train the autoregressive model is the DNA sequences clipped equally from the human reference genome. We used the standard language modeling objective $ℒ\left(S\right)=\sum_{i}\log P\left(b_{i}|b_{1},b_{2},b_{3}\ldots ,b_{i-1};\theta \right)$ to maximize the likelihood [28]. θ are the parameters of the autoregressive model that is used to model the conditional probability (See Section 2). The trained autoregressive model was used to extract features for sequencing reads. Subsequently, the extracted features from sequencing reads obtained from each individual were fed into multiple‐instance learning classifier. The attention‐based multiple‐instance classifier quantifies the influence of all instances from that individual on the classification label. Here, an instance refers to a sequencing read from that individual. Higher attention score indicates such an instance has greater discriminative capability. We trained this classifier iteratively by using cross‐entropy loss. A flow diagram describing these procedures is shown in Fig. 1.

### 3.2 High performance of DECIDIA in diagnosis of cancer

**Fig. 2:**
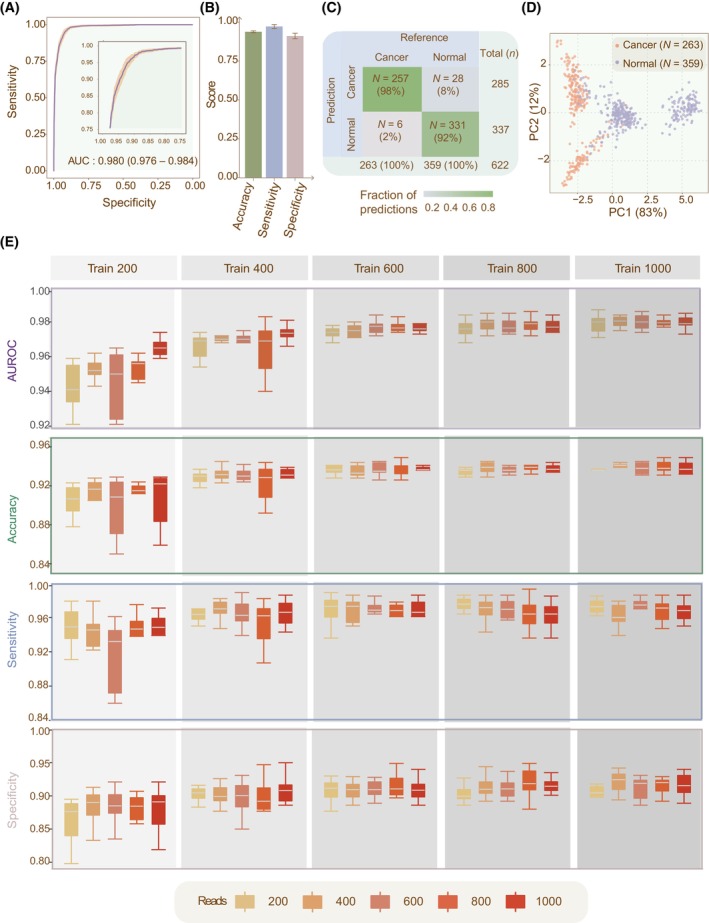
The classification performance of DECICIA in the diagnosis of cancer. (A) The ROC curve averaged from 10‐fold Monte Carlo cross‐validations for model trained with 1000 samples and each sample includes 600 reads. Insets: zoomed‐in view of the ROC curve. (B) Barplot representation of accuracy, sensitivity and specificity. (C) Confusion matrix illustrating model performance, with fractions of predictions and actual counts for cancer and normal classifications. (D) Scatter plot representation of the PCA of the learned features. (E) Boxplot representation of AUROC, accuracy, sensitivity and specificity for varying number of training samples and sequencing reads. Error bars in the barplots and boxplots indicate the 95% confidence interval. AUROC, area under the receiver operating curve; ROC, receiver operating curve.

We employed 10‐fold Monte Carlo cross‐validation [33] to evaluate the cancer diagnostic performance of DECIDIA. For each cross‐validation fold, we randomly divided the CRC dataset into training (*n* = 1000), validation (*n* = 200) and testing (*n* = 622) sets. Besides, we varied the number of training samples from 200 to 1000 and sequencing reads from 200 to 1000 for each cross‐validation fold. We trained DECIDIA on the training set and monitor its performance with validation set at the end of each training epoch. We reported the classification performance of DECIDIA on the testing set with the checkpoint that performed the best on the validation set. We observed that DECIDIA achieved equally good performance with varying number of training samples and sequencing reads (Fig. 2E). For example, DECIDIA trained with 1000 samples and 600 reads achieved an average AUROC of 0.980 (95% CI, 0.976–0.984) (Fig. 2A), accuracy of 0.938 (95% CI, 0.932–0.944), sensitivity of 0.972 (95% CI, 0.959–0.985) and specificity of 0.911 (95% CI, 0.894–0.928) (Fig. 2B,C); whereas DECIDIA trained with the minimum number of 200 training samples and 200 reads achieved an average AUROC of 0.939 (95% CI, 0.922–0.955), accuracy of 0.899 (95% CI, 0.870–0.927), sensitivity of 0.944 (95% CI, 0.910–0.977) and specificity of 0.862 (95% CI, 0.828–0.896). The performance of DECIDIA was increasing with more training samples and number of reads used. We observed that cancer patients and control individuals are distinguishable in the scatter plot generated from principal component analysis (PCA) of the features learned by DECIDIA (Fig. 2D). The other classification metrics such as positive predictive rate, negative predictive rate and F1 metric were provided in Table S2. Besides, DECIDIA also achieved better than machine learning method utilizing methylation profile reported by Luo et al. [18] (AUROC = 0.96).

**Fig. 3:**
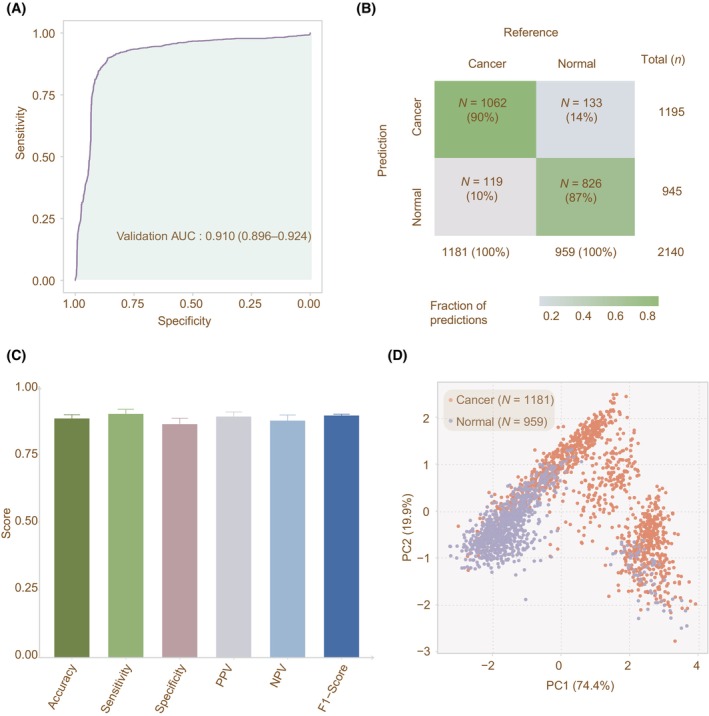
The classification performance of DECIDIA on the external HCC testing set. (A) The ROC curve depicting the model's diagnostic performance. (B) Confusion matrix illustrating model performance, with fractions of predictions and actual counts for cancer and normal classifications on the external HCC testing set. (C) Barplot representation of accuracy, sensitivity, specificity, positive predictive value, negative predictive value and F1 score. (D) Scatter plot representation of the PCA of the learned features. Error bars in the barplots indicate the 95% confidence interval. HCC, hepatocellular cell carcinoma; NPV, negative predictive value; PCA, principal component analysis; PPV, positive predictive value; ROC, receiver operating curve.

Noticeably, we observed that DECIDIA achieved an AUROC of 0.910 (95% CI, 0.896–0.924) on the externally independent hepatocellular cell carcinoma (HCC) testing set (*n* = 2140) even though no HCC data was involved in model training. The HCC testing set consists of 1181 patients with cancer and 959 controls. The ROC curve was shown in Fig. 3A. The accuracy, sensitivity and specificity achieved by DECIDIA on this HCC testing set were 0.882 (95% CI, 0.868–0.896), 0.899 (95% CI, 0.881–0.916) and 0.861 (95% CI, 0.838–0.883), respectively (Fig. 3C). Meanwhile, the PCA result from the extracted features for the HCC testing set are distinctive between HCC patients and controls (Fig. 3D).

### 3.3 High performance of DECIDIA in distinguishing different cancer types

**Fig. 4:**
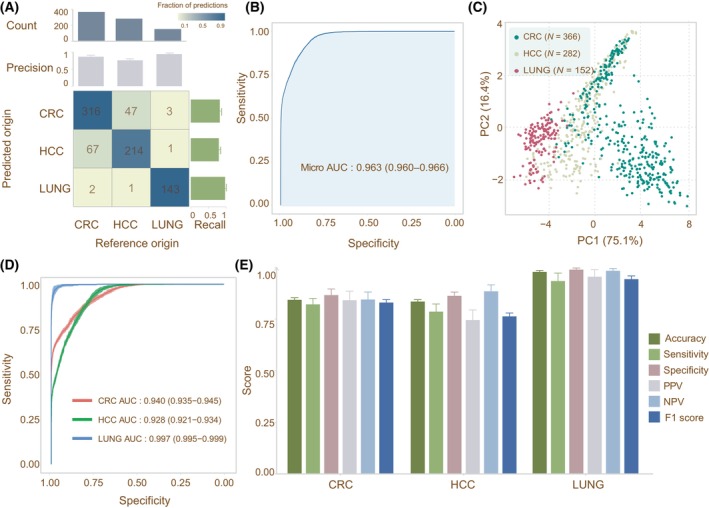
Classification performance of DECIDIA on the detection of different cancer types. (A) Confusion matrix and barplot representation of the recall and precision. (B) The micro‐average ROC curve. (C) Scatter plot representation of PCA of learned features. (D) ROC curves for the detection of each cancer type in one‐versus‐rest setting. (E) Classification performance for each cancer type. Error bars in the barplots indicate the 95% confidence interval. CRC, colorectal cancer; HCC, hepatocellular cell carcinoma; NPV, negative predictive value; PCA, principal component analysis; PPV, positive predictive value; ROC, receiver operating curve.

We curated a total number of 3409 samples for the raw targeted‐bisulfite sequencing data of plasma cfDNA from colorectal cancer (CRC, *n* = 1154), HCC (*n* = 1181) and lung cancer (*n* = 654) to evaluate the performance of DECIDIA on the detection of different cancer types. We randomly split this curated dataset into training (*n* = 2308), validation (*n* = 300) and testing (*n* = 800) sets. We used the training set to train DECIDIA and validation set to choose the best checkpoint and subsequently evaluated its performance on the testing set. On the testing set, DECIDIA achieved a micro‐averaged AUROC of 0.963 (95% CI, 0.960–0.966) and an overall accuracy of 82.8% (95% CI, 81.8–83.9%) (Fig. 4B and Table S3). Different cancer types are visually distinguishable on the scatter plot generated from the PCA of the features extracted by DECIDIA (Fig. 4C). The other classification metrics such as sensitivity, specificity and F1‐score were shown in Fig. 4E and Table S4.

### 3.4 Sequence patterns learned by DECIDIA

**Fig. 5:**
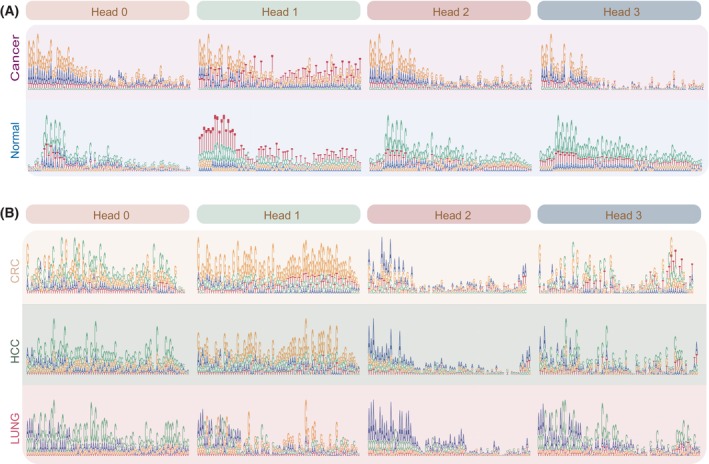
Sequence patterns learned by DECICIA. (A) Four sequence patterns in cancer versus control derived from four attention heads. (B) Sequence patterns in CRC, HCC and lung cancer derived from attention heads. The colors represent different nucleotides (A, T, C, G). The *x*‐axis represents the position within the read, and the *y*‐axis represents the attention score assigned by the model. CRC, colorectal cancer; HCC, hepatocellular cell carcinoma.

The self‐attention modules in the autoregressive model provided us a convenient way to explore the sequence patterns learned by DECIDIA (See Section 2). We constructed four sequence patterns corresponding to the four self‐attention heads of DECIDIA (See Section 2). We found distinctive sequence patterns in cancer patients versus control groups. For example, we observed that all four self‐attention heads are featured by high frequency of G in cancer group while in the control group Heads 0, 2 and 3 are featured by high frequency of C and Head 1 is featured by high frequency of T (Fig. 5A). In addition, we also observed distinctive sequence patterns among CRC, HCC and lung cancer (Fig. 5B). Specifically, Head 0 is featured by high frequency of C in HCC and lung cancer and less representation of C but higher frequency of G in CRC. Head 1 is featured by high frequency of G in CRC and HCC. Head 2 is featured by high frequency of A particularly in HCC and lung cancer. Head 3 is featured by combination of A and C in HCC and lung cancer and C, G and T in CRC.

## 4 Discussion

Accurate early cancer diagnosis is beneficial for improving prognosis [34]. In this study, we proposed a new analytical approach for cancer diagnosis exclusively from the raw sequencing reads obtained from bisulfite sequencing of the plasma cfDNA. We comprehensively evaluated this approach on four datasets for cancer diagnosis and cancer type localization. Although conceptually simple, the proposed approach eliminates the tedious data analytical procedures and is data efficient and robust.

There are numerous studies exploring the use of bisulfite sequencing of plasma cfDNA for early cancer diagnosis [18, 19]. All these studies require quantitative measurement of methylation as the first step. For example, the methylation value (also known as β‐value) for each CpG locus from bisulfite sequencing must be calculated prior to development of machine learning classifier. The β‐value is defined as the number of methylated alleles among all alleles at that CpG locus [35]. However, β‐value is reported to be insensitive if the content of tumor‐derived cfDNA is low [36]. To overcome this limitation, Stackpole and colleagues proposed the α‐value, defined as the percentage of methylated CpGs among all CpG loci in sequencing reads, to replace the β‐value [35]. However, the calculation of both α‐value and β‐value requires sequence alignment and post‐processing steps to define methylated and unmethylated cytosine. Sequence alignment of bisulfite sequencing reads is complicated and tedious [20]. Bisulfite treatment destroys the complementarity of Watson and Crick strands and leads to asymmetric distribution of C/T in that T in the sequencing reads can be aligned to C in the reference but not vice versa. A common solution is to convert all Cs to Ts for reads and map them to the converted references; subsequently, additional post‐processing steps are required to process the alignment results [37].

DECIDIA achieved at least comparable or better performance as compared with previous studies that developed machine learning classifiers on quantitative cfDNA methylation levels. In the study conducted by Luo et al. [18], they reported an AUROC of 0.96, sensitivity of 87.9% and specificity of 89.6% in diagnosis of CRC with random forest [38] classifier by using the differential methylation markers. In contrast, DECIDIA achieved an AUROC of 0.98, sensitivity of 0.972 and specificity of 0.911, underscoring the high classification performance.

DECIDIA is data efficient. We demonstrated this advantage with varying number of samples and sequencing reads ranged from 200 to 1000 with a step size of 200. We observed that the performance of DECIDIA on the minimum data size versus maximum data size are marginal. DECIDIA achieved an AUROC of 0.939 when it was developed with 200 samples and 200 reads and an AUROC of 0.980 when it was developed with 1000 samples and 600 reads (Fig. 2E and Table S2).

The performance of DECIDIA is robust. This is exemplified by the high performance of our approach on the independent HCC testing set. Although the HCC testing cohort was not involved in the model development, DECIDIA achieved an AUROC of 0.910 on this HCC testing set. This result suggested that there is shared methylated cfDNA patterns among different cancer types and DECIDIA succeeded to learn these shared features. We indeed observed similar patterns of methylated cfDNA from the CRC and HCC datasets (Fig. 5B).

The use of raw sequencing reads confers several advantages over previous studies that relies on the quantitative cfDNA methylation abundance. Firstly, our solution might be more sensitive towards samples with extremely low content of plasma cfDNA in that precise quantification of tumor methylation levels from low content cfDNA is error‐prone [12]. Secondly, the methylation signatures of a single cfDNA fragment might be insensitive towards batch effect as compared with quantitative cfDNA methylation abundance. Additional analysis indicates that our method is insensitive to batch effect (Fig. S1). However, traditional bioinformatics analyses are more likely to introduce batch effect during sequence alignment and quantification of methylation abundance [39, 40]. Thirdly, our approach is intuitively and conceptually simple as it does not require base conversion of the sequencing reads and sequence alignment, leading to faster development of diagnostic model and prediction once deployed. In addition, our approach can use as diverse as DNA fragments from the complete cfDNA repertoire whereas alignment‐based approaches may discard the exogeneous DNA fragments if they cannot be mapped onto the human genome reference. The exogeneous DNA fragments deriving from bacteria and virus have been demonstrated to be helpful for cancer detection [41]. These merits of our approach would revolutionize the way of plasma cfDNA methylation being used in cancer diagnosis.

However, our approach was not without limitations. The DNA sequence representation learning model was developed exclusively with the human reference genome but not bisulfite sequencing reads. Given that bisulfite treatment changes the unmethylated cytosine to thymine, the sequence patterns of methylated DNA fragments from bisulfite sequencing would be different the reference genome sequence; therefore, features of bisulfite sequencing reads learned by the model developed with human reference genome would be suboptimal. The performance of our approach would be boosted if the feature representation model of DNA sequences were developed with the raw bisulfite sequencing reads. In addition, the weakly supervised learning model used for classification does not model the inter‐dependence among different reads but treat them independently. Improvement of the algorithm allowing it to automatically model inter‐dependence of sequencing reads has the potential to increase the performance of the diagnostic model. We will investigate the aforementioned limitations in future studies. In addition, the TNM staging information is not available from these published studies [18, 19, 27] where the sequencing data were collected. Therefore, so we are not able to perform subgroup analysis in terms of TNM stages. Besides, the number of cancer types used for evaluation of cancer type localization are limited given that the freely available raw sequencing data of plasma cfDNA subjected to bisulfite sequencing is still limited.

Although the model developed on CRC dataset achieved high accuracy in detection of cancer when it was evaluated on the HCC dataset, we cannot rule out the possibility of batch effect that may compromise the results. Notably, we observed that sequencing reads from these two datasets projected by PCA are well admixed (Fig. S1), suggesting that impact of batch effect is minimal. While cfDNA derived from cancer cells in plasma is a well‐established direct marker for cancer, we propose that cfDNA fragments originating from non‐cancerous cells in cancer patients may also provide valuable insights for cancer detection. The cfDNA fragmentome reveals variations in nucleosome topology [42] and diverse nucleosome organizations [43, 44] among different cell types. We hypothesize that physiological alterations associated with cancer could impact nucleosome topology in non‐cancerous cells, resulting in distinct topological signatures that differ from those observed in cancer cells. These unique topological structures in non‐cancerous cells may offer additional signals for cancer detection [45, 46]. Furthermore, the datasets utilized in our study were subjected to enrichment based on differential methylation patterns between cancerous tissues and adjacent normal controls. This enrichment process substantially amplifies the cancer signal. Consequently, we have the potential to achieve high sensitivity and specificity in cancer detection using a relatively small number of reads. Future studies are essential to explore and validate this hypothesis and to address any potential limitations in the datasets.

Apart from the discrimination between cancer patients from control individuals, our approach is able to identify sequencing reads that are most relevant for classification. Analyses of the most relevant reads lead us to identify distinctive motifs in cancer versus control groups and analogous motifs among different cancer types.

The diagnostic model developed in our study is light‐weighted in that it can be run locally on the web browser. We set up a website to provide free access to the developed model for testing and benchmarking purpose. The website is freely available at http://lixiangchun.github.io/DECIDIA/index.html.

## 5 Conclusions

In conclusion, we herein reported a deep‐learning approach for early cancer diagnosis exclusively from the raw sequencing reads of plasma cfDNA subjected to bisulfite sequencing. DECIDIA eliminates the complicated and tedious data analytical procedures. DECIDIA represents a new paradigm of diagnostic model development for liquid biopsy.

## Conflict of interest

The authors declare no conflict of interest.

## Author contributions

X.L. and K.C. contributed to the conception and design of the study; J.L., H.S., Y.Y., M.Y., and Q.Z. contributed to the acquisition of data and/or analysis, curation, and interpretation of data; X.L., K.C., J.L., and H.S. contributed to the generation of important models and the contribution of essential resources, technology, and funding; X.L., J.L., and H.S. contributed to drafting the manuscript; all authors contributed to the revision of the manuscript and approved the final version for publication.

## Supporting information


**Fig. S1.** PCA visualization of read features from CRC(PRJNA574555) and HCC(PRJNA360288) datasets.
**Table S1.** Data source information.
**Table S2.** Classification metrics for different number of samples and sequencing reads.
**Table S3.** Overall accuracy for multi‐cancer classification in ten‐fold cross‐validation setting.
**Table S4.** Classification metrics stratified by cancer types.
**Table S5.** Detailed experiments protocol for datasets in this study.

## Data Availability

The authors declare that the data supporting the findings of this study are available within the paper and its Supporting Information files. Raw sequencing data was downloaded from Sequence Read Archive (SRA) with the Accession Number PRJNA574555 [18], PRJNA360288 [19], PRJNA383358 [27] and PRJNA383370 [27]. Code is available at https://github.com/deeplearningplus/DECIDIA.
