# Supplementary material for: Transformer‐based representation learning and multiple‐instance learning for cancer diagnosis exclusively from raw sequencing fragments of bisulfite‐treated plasma cell‐free DNA
Source: Mol Oncol. 2024 Oct 8;18(11):2755–69. doi: 10.1002/1878-0261.13745 (PMC11547222; doi:10.1002/1878-0261.13745)
Supplement: Supplementary file 1 — Fig. S1. PCA visualization of read features from CRC(PRJNA574555) and HCC(PRJNA360288) datasets. Table S1. Data source information. Table S2. Classification metrics for different number of samples and sequencing reads. Table S3. Overall accuracy for multi‐cancer classification in ten‐fold cross‐validation setting. Table S4. Classification metrics stratified by cancer types. Table S5. Detailed experiments protocol for datasets in this study. [file MOL2-18-2755-s001.docx]

**Supplementary materials**


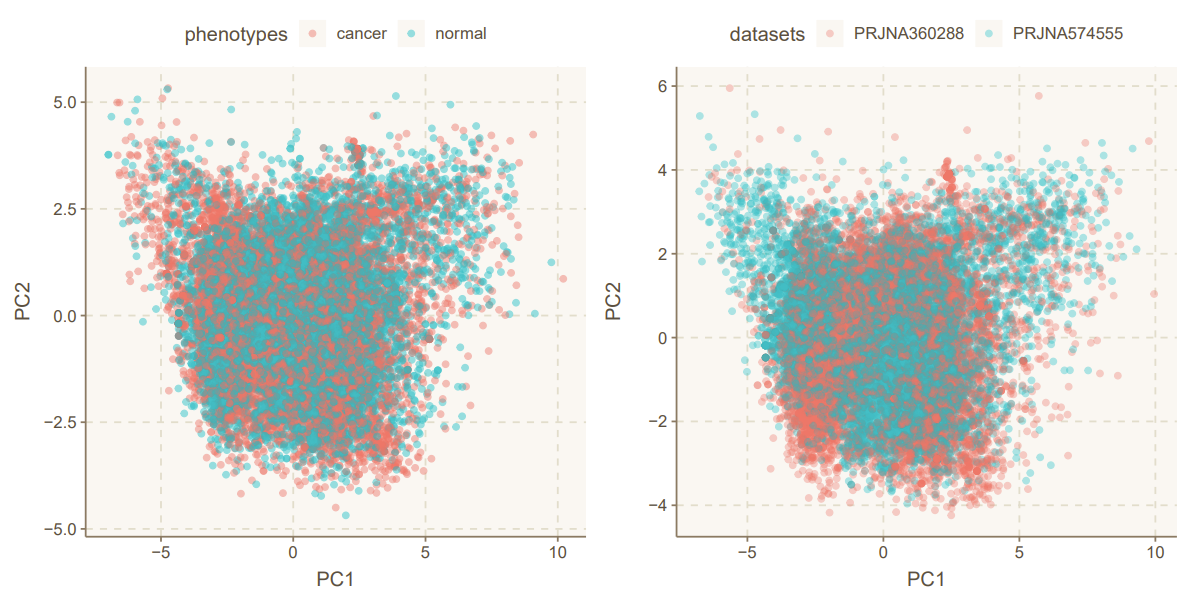


**Supplementary Figure 1. PCA visualization of read features from CRC (PRJNA574555) and HCC (PRJNA360288) datasets.**

**Supplementary Table 1. Data source information**

| SRA accession ID | Total samples | Cancer samples | Control samples | Reference | Description |
| --- | --- | --- | --- | --- | --- |
| PRJNA574555 | 1822 | 801 | 1021 | http://www.ncbi.nlm.nih.gov/bioproject/574555 | cfDNA methylation for screening and surveillance of colorectal cancer |
| PRJNA360288 | 2140 | 1181 | 959 | https://www.ncbi.nlm.nih.gov/bioproject/?term=PRJNA360288 | ctDNA methylation markers in diagnosis and prognosis of hepatocellular carcinoma |
| PRJNA383358 | 773 | 773 | NA | https://www.ncbi.nlm.nih.gov/bioproject/?term=PRJNA383358 | Cell-free DNA methylation markers in diagnosis and prognosis of common cancers (COAD) |
| PRJNA383370 | 654 | 654 | NA | https://www.ncbi.nlm.nih.gov/bioproject/?term=PRJNA383370 | Cell-free DNA methylation markers in diagnosis and prognosis of common cancers (lung cancer) |

**Supplementary Table 2. Classification metrics for different number of samples and sequencing reads**

| Number of reads per sample | | Number of samples | Accuracy | Sensitivity | Specificity | Positive predictive value | Negative predictive value | F1-score |
| --- | --- | --- | --- | --- | --- | --- | --- | --- |
| 1000 | 1000 | | 0.939 ± 0.0047 | 0.967 ± 0.0080 | 0.916 ± 0.0118 | 0.903 ± 0.0120 | 0.972 ± 0.0069 | 0.934 ± 0.0044 |
| 1000 | 200 | | 0.907 ± 0.0203 | 0.941 ± 0.0189 | 0.880 ± 0.0253 | 0.863 ± 0.0290 | 0.949 ± 0.0160 | 0.900 ± 0.0222 |
| 1000 | 400 | | 0.934 ± 0.0049 | 0.966 ± 0.0106 | 0.909 ± 0.0150 | 0.895 ± 0.0153 | 0.971 ± 0.0090 | 0.929 ± 0.0048 |
| 1000 | 600 | | 0.933 ± 0.0105 | 0.962 ± 0.0181 | 0.909 ± 0.0109 | 0.895 ± 0.0143 | 0.968 ± 0.0138 | 0.927 ± 0.0130 |
| 1000 | 800 | | 0.933 ± 0.0093 | 0.961 ± 0.0138 | 0.911 ± 0.0168 | 0.897 ± 0.0157 | 0.966 ± 0.0120 | 0.927 ± 0.0088 |
| 200 | 1000 | | 0.936 ± 0.0022 | 0.973 ± 0.0055 | 0.907 ± 0.0054 | 0.893 ± 0.0069 | 0.976 ± 0.0049 | 0.931 ± 0.0029 |
| 200 | 200 | | 0.899 ± 0.0202 | 0.944 ± 0.0239 | 0.862 ± 0.0243 | 0.847 ± 0.0233 | 0.950 ± 0.0211 | 0.893 ± 0.0210 |
| 200 | 400 | | 0.929 ± 0.0042 | 0.962 ± 0.0053 | 0.903 ± 0.0077 | 0.888 ± 0.0068 | 0.967 ± 0.0041 | 0.923 ± 0.0044 |
| 200 | 600 | | 0.936 ± 0.0035 | 0.969 ± 0.0116 | 0.909 ± 0.0117 | 0.896 ± 0.0102 | 0.974 ± 0.0094 | 0.931 ± 0.0033 |
| 200 | 800 | | 0.936 ± 0.0042 | 0.974 ± 0.0076 | 0.905 ± 0.0106 | 0.892 ± 0.0097 | 0.977 ± 0.0061 | 0.931 ± 0.0038 |
| 400 | 1000 | | 0.940 ± 0.0026 | 0.963 ± 0.0091 | 0.921 ± 0.0110 | 0.908 ± 0.0103 | 0.969 ± 0.0072 | 0.935 ± 0.0020 |
| 400 | 200 | | 0.905 ± 0.0203 | 0.935 ± 0.0245 | 0.881 ± 0.0202 | 0.864 ± 0.0232 | 0.944 ± 0.0214 | 0.898 ± 0.0220 |
| 400 | 400 | | 0.932 ± 0.0047 | 0.969 ± 0.0087 | 0.902 ± 0.0112 | 0.888 ± 0.0119 | 0.973 ± 0.0070 | 0.927 ± 0.0054 |
| 400 | 600 | | 0.932 ± 0.0095 | 0.962 ± 0.0191 | 0.908 ± 0.0097 | 0.893 ± 0.0114 | 0.968 ± 0.0142 | 0.926 ± 0.0121 |
| 400 | 800 | | 0.938 ± 0.0041 | 0.969 ± 0.0096 | 0.914 ± 0.0124 | 0.901 ± 0.0129 | 0.974 ± 0.0075 | 0.933 ± 0.0040 |
| 600 | 1000 | | 0.938 ± 0.0043 | 0.972 ± 0.0091 | 0.911 ± 0.0123 | 0.898 ± 0.0114 | 0.976 ± 0.0076 | 0.933 ± 0.0038 |
| 600 | 200 | | 0.897 ± 0.0219 | 0.914 ± 0.0290 | 0.884 ± 0.0195 | 0.863 ± 0.0263 | 0.929 ± 0.0223 | 0.888 ± 0.0258 |
| 600 | 400 | | 0.927 ± 0.0109 | 0.965 ± 0.0117 | 0.898 ± 0.0175 | 0.883 ± 0.0203 | 0.970 ± 0.0095 | 0.922 ± 0.0124 |
| 600 | 600 | | 0.939 ± 0.0063 | 0.971 ± 0.0047 | 0.913 ± 0.0131 | 0.900 ± 0.0137 | 0.975 ± 0.0043 | 0.934 ± 0.0062 |
| 600 | 800 | | 0.934 ± 0.0093 | 0.961 ± 0.0247 | 0.911 ± 0.0098 | 0.897 ± 0.0107 | 0.968 ± 0.0173 | 0.927 ± 0.0127 |
| 800 | 1000 | | 0.940 ± 0.0045 | 0.968 ± 0.0093 | 0.917 ± 0.0119 | 0.904 ± 0.0115 | 0.973 ± 0.0072 | 0.935 ± 0.0043 |
| 800 | 200 | | 0.907 ± 0.0163 | 0.937 ± 0.0258 | 0.883 ± 0.0135 | 0.865 ± 0.0187 | 0.947 ± 0.0195 | 0.899 ± 0.0201 |
| 800 | 400 | | 0.922 ± 0.0137 | 0.952 ± 0.0196 | 0.898 ± 0.0166 | 0.883 ± 0.0174 | 0.959 ± 0.0170 | 0.916 ± 0.0144 |
| 800 | 600 | | 0.938 ± 0.0061 | 0.965 ± 0.0096 | 0.916 ± 0.0135 | 0.903 ± 0.0142 | 0.970 ± 0.0085 | 0.933 ± 0.0059 |
| 800 | 800 | | 0.940 ± 0.0045 | 0.964 ± 0.0125 | 0.920 ± 0.0150 | 0.907 ± 0.0148 | 0.969 ± 0.0104 | 0.934 ± 0.0040 |

**Values are mean ± 95%CI calculated from the results of the cross-validation folds.*

**Supplementary Table 3. Overall accuracy for multi-cancer classification in ten-fold cross-validation setting**

| Fold | Overall accuracy |
| --- | --- |
| 0 | 0.815 (95%CI, 0.786-0.841) |
| 1 | 0.841 (95%CI, 0.814-0.866) |
| 2 | 0.826 (95%CI, 0.798-0.852) |
| 3 | 0.819 (95%CI, 0.790-0.845) |
| 4 | 0.834 (95%CI, 0.806-0.859) |
| 5 | 0.836 (95%CI, 0.809-0.861) |
| 6 | 0.838 (95%CI, 0.810-0.862) |
| 7 | 0.833 (95%CI, 0.805-0.858) |
| 8 | 0.810 (95%CI, 0.781-0.837) |
| 9 | 0.831 (95%CI, 0.803-0.857) |

**Supplementary Table 4. Classification metrics stratified by cancer types**

| Group | Accuracy | Sensitivity | Specificity | Positive predictive value | Negative predictive value | F1-score |
| --- | --- | --- | --- | --- | --- | --- |
| Colorectal cancer | 0.843 ± 0.0074 | 0.821 ± 0.0208 | 0.866 ± 0.0213 | 0.841 ± 0.0313 | 0.845 ± 0.0259 | 0.830 ± 0.0104 |
| Hepatocellular carcinoma | 0.835 ± 0.0067 | 0.786 ± 0.0266 | 0.863 ± 0.0130 | 0.745 ± 0.0352 | 0.884 ± 0.0223 | 0.763 ± 0.0118 |
| Lung cancer | 0.978 ± 0.0048 | 0.934 ± 0.0277 | 0.990 ± 0.0055 | 0.955 ± 0.0245 | 0.984 ± 0.0078 | 0.943 ± 0.0113 |

**Values are mean ± 95%CI calculated from the results of the cross-validation folds*

**Supplementary Table 5. Detailed experiments protocol for datasets in this study**

|  | PRJNA574555 | PRJNA360288 | PRJNA383358 | PRJNA383370 |
| --- | --- | --- | --- | --- |
| Input plasma cfDNA | 1.5ml | 1.5ml | 1.5ml | 1.5ml |
| extraction kit | EliteHealth® | EliteHealth® | EliteHealth® | EliteHealth® |
| Methylation convert kit | EZ DNA Methylation-Lightning Kit (Zymo Research®) | EZ DNA Methylation-Lightning Kit (Zymo Research®) | EZ DNA Methylation-Lightning Kit (Zymo Research®) | EZ DNA Methylation-Lightning Kit (Zymo Research®) |
| Probe design software | ppDesigner | ppDesigner | ppDesigner | ppDesigner |
| Sequencing platform | Illumina HiSeq 2500® | Illumina HiSeq 2500® | Illumina HiSeq 2500® | Illumina HiSeq 2500® |
| Layout | Paired | Paired | Paired | Paired |
| Reads length | 150 | 75 | 150 | 150 |
| Total reads | (0.107-3.33)*10^6^ | (0.110-2.21)*10^6^ | (0.104-1.83)*10^6^ | (0.497-2.08)*10^6^ |
